# Low-grade fibromyxoid sarcoma of the external anal sphincter: a case report

**DOI:** 10.1186/s12957-017-1174-8

**Published:** 2017-05-30

**Authors:** Lee-Kiat Ban, Ailun Heather Tseng, Shih-Hung Huang, Henry Hsin-Chung Lee

**Affiliations:** 1 Department of Surgery, Hsinchu Cathay General Hospital, Jhonghua Rd, Hsinchu, Taiwan; 2 College of Medical Technology, Nursing and Wellbeing, Yuanpei University of Medical Technology, Yuanpei Street, Hsinchu, Taiwan; 3 Department of Pathology, Cathay General Hospital, Jen-Ai Road, Taipei, Taiwan; 4 Graduate Institute of Translational and Interdisciplinary Medicine, College of Health Sciences and Technology, National Central University, Jung-Da Rd, Taoyuan, Taiwan; 5 School of Medicine, Fu Jen Catholic University, Zhongzheng Rd, New Taipei, Taiwan

**Keywords:** Low-grade fibromyxoid sarcoma, LGFMS, External anal sphincter, Perineum

## Abstract

**Background:**

Low-grade fibromyxoid sarcoma (LGFMS) is a rare soft tissue tumor that has a tendency to grow in the deep soft tissue of the trunk and extremities. Despite its benign appearance, the tumor has a high recurrence rate and metastatic potential. LGFMS in the perineal space is rare, and only a few cases have been reported. We present the first case of LGFMS to be located at the external anal sphincter.

**Case presentation:**

A 27-year-old male patient admitted to our Surgical Department with perianal pain and swollen for a year. The digital rectal examination revealed a perianal mass. Oral metronidazole and analgesia were prescribed on suspicion of perianal abscess failed to alleviate the symptom; hence, the patient was scheduled for surgery. Intraoperative diagnosis revealed an encapsulated tumor in the external anal sphincter that extended from the perianal region orally to the pararectal space. The results of immunohistochemistry (MUC4 staining) and *FUS* gene rearrangement by fluorescence in situ hybridization confirmed the diagnosis of LGFMS.

**Conclusions:**

This case is unique in terms of the location of the rare soft tissue tumor. Although LGFMS is considered low grade, its unpredictable behavior necessitates a long-term follow-up.

## Background

Low-grade fibromyxoid sarcoma (LGFMS), a rare soft tissue tumor, was first described by Evans in 1987 [1]. Despite its deceptively benign appearance, LGFMS has a high tendency for local recurrence and late distant metastasis, mainly to the lung [2, 3]. The histological appearance of LGFMS is characterized by the presence of alternating fibrous and myxoid area in a whorled growth pattern [3]. Although LGFMS is common in middle-aged adults, 13–19% of cases occur in 18 years and younger. To date, the youngest reported case was 22-month-old [2, 4, 5].

Given its bland appearance, LGFMS can be difficult to distinguish from some benign mesenchymal tumors and other low-grade sarcomas [3]. Epithelial membrane antigen (EMA) is one of the diagnostic markers for LGFMS, but the poor specificity can also be detected in soft tissue perineurioma and a subset of solitary fibrous tumors [6–8]. Recently, Doyle et al. found that mucin 4 (*MUC4*) was a highly specific and sensitive marker for LGFMS when compared with other histologically similar tumors [3]. Furthermore, *FUS* gene rearrangement by fluorescence in situ hybridization (FISH) and *FUS-CREB3L2/FUS-CREB3L1* chimeric fusion genes by reverse transcription polymerase chain reaction are the other two reliable approaches for LGFMS diagnosis [9].

We present the first case of a 27-year-old male with LGFMS in the external anal sphincter. The diagnosis of LGFMS was confirmed by MUC4 staining and *FUS* gene rearrangement using immunohistochemistry (IHC) and FISH, respectively.

## Case presentation

A 27-year-old male with no relevant medical or surgical history who presented to the Surgical Department complaining of intermittent perianal pain for a year. He described a normal bowel habit of two to three times a day but rectal bleeding following defecation for 1 week. Physical examinations were unremarkable except a considerable redness, swelling, and tenderness on palpation over the perianal region. Digital rectal examination revealed a perianal tender mass. A prescription of oral metronidazole and analgesia for 1 week due to suspicion of perianal abscess failed to alleviate the symptom; hence, the patient was scheduled for surgical intervention. Intraoperative diagnosis revealed an encapsulated tumor in the external anal sphincter that extended from the perianal region orally to the pararectal space and there were no other abnormalities. The tumor was enucleated for definitive diagnosis.

**Fig. 1:**
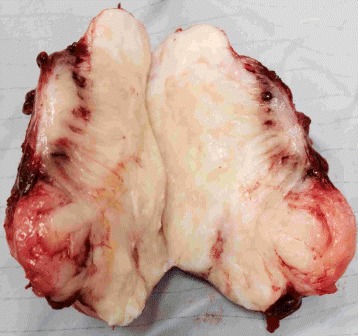
Gross morphology of LGFMS from the 27-year-old patient. The resected tumor from the external anal sphincter is tan, firm, and has a glistening appearance

**Fig. 2:**
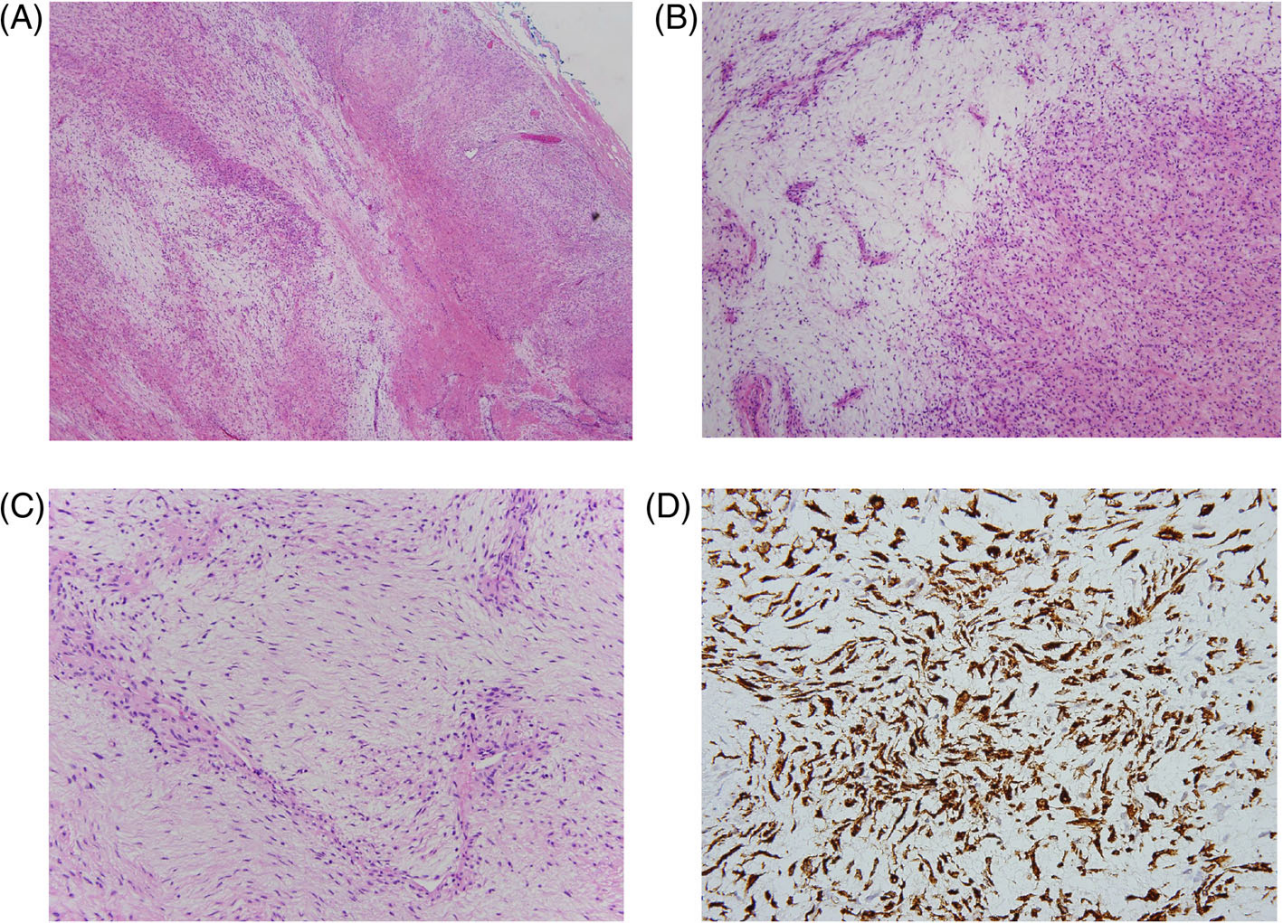
Classic histological features of the LGFMS. Histological images of the LGFMS show **a** an admixture of collagenized and hypocellular myxoid zones (H&E, ×40), **b** alternating hypocellular myxoid areas of whorled growth patterns (H&E, ×100), **c** bland-appearing small uniform spindle cells with arcades of small vessels (H&E, ×200), and **d** strong immunoreactivity of MUC4 (×400)

**Fig. 3:**
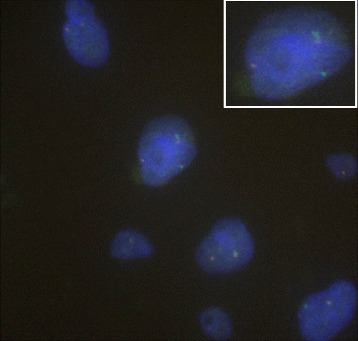
Coexistence of fused- and split-signals of the FUS gene. FISH analysis of FUS (16p11) using dual-color probes detected both fused- and split-signals

The gross specimen, measuring 6.1×4.6×4.3 cm, was tan and firm in appearance (Fig. 1). IHC analysis of the specimen showed an alternating myxoid and densely collagenous area with low-grade spindle cells (Fig. 2 a–c). MUC4 stain (Fig. 2 d) and *FUS* gene rearrangement by FISH (Fig. 3) were subsequently ordered for suspicion of LGFMS and all came back positive. The findings confirmed the diagnosis of LGFMS.

The patient underwent regular clinical follow-ups at 3-month interval and CT scan annually. One year after surgery, the patient was asymptomatic and in good health. The external anal sphincter muscle was fully functional, and the CT scan revealed no abnormal findings.

## Discussion

The anatomic locations of LGFMS are common in the soft tissues of the upper limbs, lower limbs, and trunk wall [2, 5]. LGFMS occurring in the perineal space is extremely rare and only a few cases have been reported [10–17]. Park et al. published the first LGFMS in the colon of a 43-year-old man whose tumor was surgically removed along with the creation of right hemicolectomy combined with nephrectomy. The patient recovered well 24 months after surgery [17]. In another case, Mendoza and colleagues recently reported a 48-year-old female with LGFMS that arose from the sigmoid colon without metastatic disease. Despite the patient later developed a bowel obstruction that required another surgery, she ultimately recovered and was discharged to home; however, the follow-up information is not available [10]. Although LGFMS has a relatively good prognosis, the rate of local recurrence and late metastasis are high [15]. In 2011, Evans demonstrated an extensive follow-up study of 33 LGFMS cases with metastases. Of the 33 cases examined, 15 of them metastasized mostly to the lungs, pleura and chest wall, and occasionally to the pericardium. Other sites were also noted (two bones, one liver, and one heart). The interval to distant metastases varied up to 45 years with a median of 5 years, suggesting the indolent nature of the tumor [12].

The dilemma of LGFMS diagnosis lies on its benign appearance. IHC is a useful tool for diagnosis, but the markers are of limited values as the staining profile of LGFMS is nonspecific. For instance, we examined several markers such as desmin, S-100, CD34, and EMA, but the results were negative (data not shown). The findings are consistent with the published literatures [2, 10, 15]. Doyle and colleagues identified MUC4 as a highly sensitive and specific marker for LGFMS through gene expression profiling. To confirm the finding, 49 LGFMS cases were evaluated and all exhibited strong and diffuse cytoplasmic staining pattern [3]. However, when applied to suspicious MUC4-negative cases of LGFMS, *FUS* gene rearrangement should be exploited to guide the diagnosis [9, 18].

Surgical resection remains the standard treatment for LGFMS, with wide en bloc surgical resection being the most effective [5, 19] and enucleation, which was performed, being optimal for eliminating functional impairment of the anal sphincter muscle. We performed no adjuvant treatments because such practices to LGFMS are not conventional. Radiotherapy has effectiveness that remains in question to date and is performed only when factors such as margin positivity, tumor location, and tumor size indicate that recurrence or metastasis is likely. Chemotherapy is reserved for patients whose tumors recur locally or spread to distant sites [5, 20–22]. Overall, optimal treatment plans for maximizing oncologic control and delivering least functional impairments often involve multidisciplinary discussions.

## Conclusions

This case report enriches the literature with information on the site of LGFMS. Although the site of occurrence and benign appearance of LGFMS often make diagnosis difficult, the advancement in molecular pathology has proved to be useful in clinical practices. Finally, the rate of occurrence and metastasis are high, and long-term follow-up is prudent.

## Abbreviations

EMA: Epithelial membrane antigen
FISH: Fluorescence in situ hybridization
IHC: Immunohistochemistry
LGFMS: Low-grade fibromyxoid sarcoma
MUC4: Mucin 4
